# From equator to pole: splitting chromosomes in mitosis and meiosis

**DOI:** 10.1101/gad.255554.114

**Published:** 2015-01-15

**Authors:** Eris Duro, Adèle L. Marston

**Affiliations:** The Wellcome Trust Centre for Cell Biology, Institute of Cell Biology, School of Biological Sciences, University of Edinburgh, Edinburgh EH9 3BF, United Kingdom

**Keywords:** kinetochore, meiosis, microtubules, mitosis

## Abstract

During eukaryotic cell division, chromosomes must be precisely partitioned to daughter cells. This relies on a mechanism to move chromosomes in defined directions within the parental cell. While sister chromatids are segregated from one another in mitosis and meiosis II, specific adaptations enable the segregation of homologous chromosomes during meiosis I to reduce ploidy for gamete production. Many of the factors that drive these directed chromosome movements are known, and their molecular mechanism has started to be uncovered. Here we review the mechanisms of eukaryotic chromosome segregation, with a particular emphasis on the modifications that ensure the segregation of homologous chromosomes during meiosis I.

## Segregation machinery and components

The accurate segregation of the genetic material in eukaryotes is guided by three basic principles: (1) Force needs to be generated to power the movement of the DNA. (2) DNA needs to be linked to other cellular structures that will mediate its segregation. (3) The units of DNA to be partitioned need to be held together prior to being segregated. The molecular components that ensure that these requirements are fulfilled are described below.

### Powering chromosome movement (microtubules)

**Figure 1.**
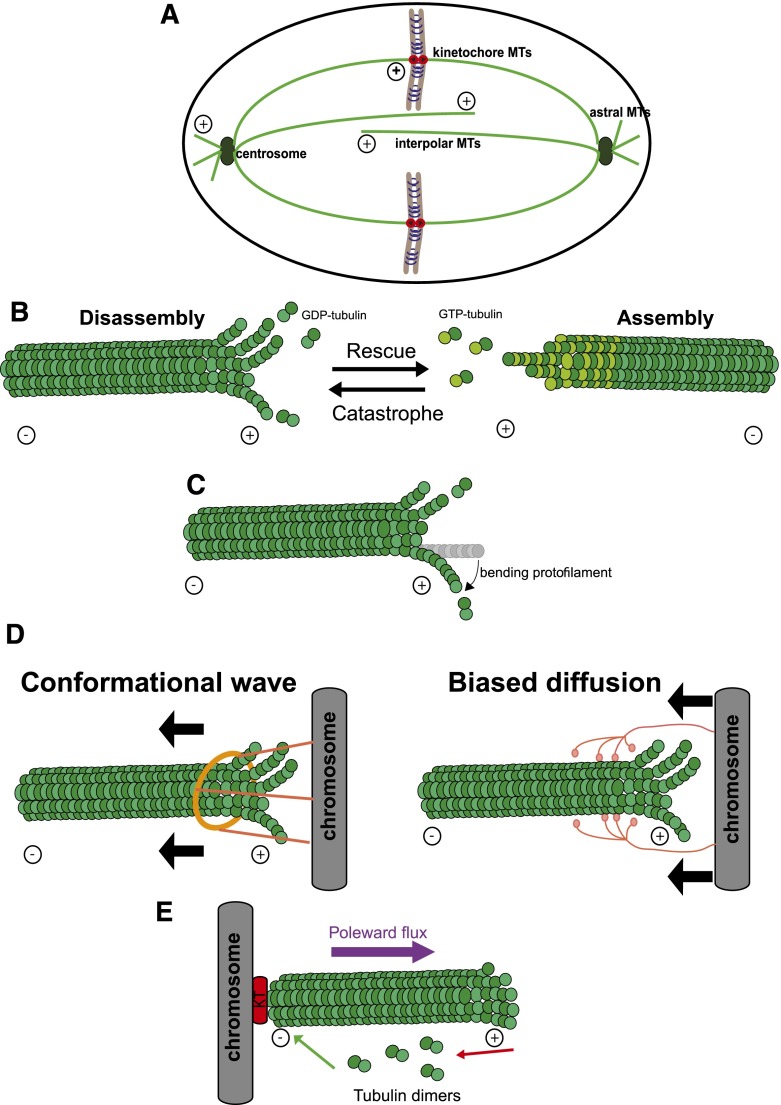
Microtubules drive chromosome motion. (*A*) The different types of microtubules (light green) nucleated by the centrosome (dark green): Astral microtubules project into the cell cortex, kinetochore microtubules connect the poles to the chromosomes to be segregated, and interpolar microtubules interdigitate to provide structural rigidity. (*B*) The coexistence of assembly and disassembly at the plus end of microtubules is described as dynamic instability. Microtubules grow via the addition of GTP-bound α-tubulin and β-tubulin dimers. (*C*) The energy released from GTP hydrolysis during microtubule assembly is stored in the polymer lattice via the geometrical constraint imposed by the bend. (*D*) Two different models of how microtubule depolymerization can provide the energy for directional motion of chromosomes. (*Left* panel) In the conformational wave model, as the disassembling protofilaments curve outward, a “sliding collar” (often posited to be a ring; see the text) is driven toward the minus end. (*Right* panel) In the biased diffusion model, a binding free-energy gradient ensures biased direction. (*E*) Addition of tubulin subunits to kinetochore-bound microtubule plus ends counteracts the loss of tubulin subunits from the minus ends, thus creating a constant poleward flow of tubulin subunits. This poleward flux is thought to contribute to correct microtubule attachment and chromosome motion.

The most prominent structure in a mitotic cell is the bipolar spindle (made up of microtubules and associated motor proteins), which provides the force to move chromosomes and thereby bring about their segregation. Microtubules are nucleated by the centrosome (called spindle pole body [SPB] in yeasts). Microtubules are assembled from heterodimers of α-tubulin and β-tubulin, which self-assemble in their GTP-bound state into rigid tubes with an ∼25-nm outside diameter, the walls of which are built from a single layer of tubulins (Fig. 1; Desai and Mitchison 1997). Microtubules are polar, with their minus end at or near the spindle pole, and the plus end projecting away from the spindle pole (Fig. 1A). One feature that underpins the biological role of microtubules (see below) is that they are dynamic; i.e., new subunits can be added or removed from either end. They can switch from polymerization to depolymerization (catastrophe) or vice versa (rescue) (Fig. 1B) in response to GTP hydrolysis within the tubulin dimers themselves as well as the activity of associated motor proteins and regulators.

Given their inherent dynamics and the existence of associated motor proteins, microtubules could theoretically contribute to chromosome segregation by acting in two ways: as a ratchet to exert pushing and pulling forces or as tracks along which cellular motors can carry chromosomes as cargo. Although motors play important roles in chromosome segregation (Nicklas 1989; Song and Mandelkow 1993; Endow et al. 1994; Noda et al. 1995; Gaglio et al. 1996; Tytell and Sorger 2006), they are not essential in fungi (Tanaka et al. 2005, 2007; Grishchuk and McIntosh 2006), and their depletion in vertebrates does not completely abolish chromosome motion (Kapoor et al. 2006; Yang et al. 2007). Additionally, microtubules can support directional motion in the absence of motor function (Koshland et al. 1988; Lombillo et al. 1995; Grishchuk et al. 2005). Indeed, microtubule depolymerization is thought to provide the primary force that drives chromosome motion: A single depolymerizing microtubule can generate up to 10 times as much force as a motor enzyme (Inoué and Salmon 1995). Microtubules grow via the addition of GTP-bound tubulin dimers, which hydrolyze GTP after polymerization. The GDP-bound dimer is bent compared with the GTP-bound counterpart (Fig. 1C). This bend is constrained within the microtubule lattice in such a way that some of the energy released from GTP hydrolysis is stored in the polymer lattice in the form of physical strain. During microtubule depolymerization, this energy is released as the dissociated tubulin dimers adopt their preferred bent conformation. It has been estimated that a single protofilament can generate up to 5 pN during depolymerization; this means that a single microtubule (composed of 13 protofilaments) can produce a force of 65 pN (Grishchuk et al. 2005). This is far higher than what is required for chromosome segregation—as little as 0.1pN, as predicted by theoretical analyses (Nicklas 1965). In a pioneering study, Nicklas (1983) was able to measure the force exerted by the spindle on a single chromosome. By using a microneedle to apply and measure the force needed to stall a chromosome in grasshopper spermatocytes, he estimated that the spindle could generate up to 700 pN on a single kinetochore with multiple microtubules attached (Nicklas 1983). However, more recent measurements of spindle forces suggest that this might be a large overestimation (Ferraro-Gideon et al. 2013). Thus, it is still uncertain how much force a spindle generates in cells.

### Linking chromosomes to the spindle (kinetochores)

Harnessing the energy provided by microtubules and converting it into directional and processive chromosome movement require a coupling device that can associate with both chromosomes and microtubules while resisting the force applied on chromosomes. The kinetochore is the structure that achieves this feat. Kinetochores are protein complexes that assemble on centromeres, specific regions of each chromosome specified by the presence of the histone H3 variant CENP-A. Kinetochore function depends on their ability to form persistent load-bearing attachments to the highly dynamic plus ends of microtubules. The persistent attachment is important, since, due to lack of inertia in the cellular environment, force needs to be constantly applied on chromosomes during their separation. Our understanding of the properties and function of the kinetochore has been enhanced by several lines of investigation: from genetics and proteomics to structural biology and single-molecule biophysics.

**Figure 2.**
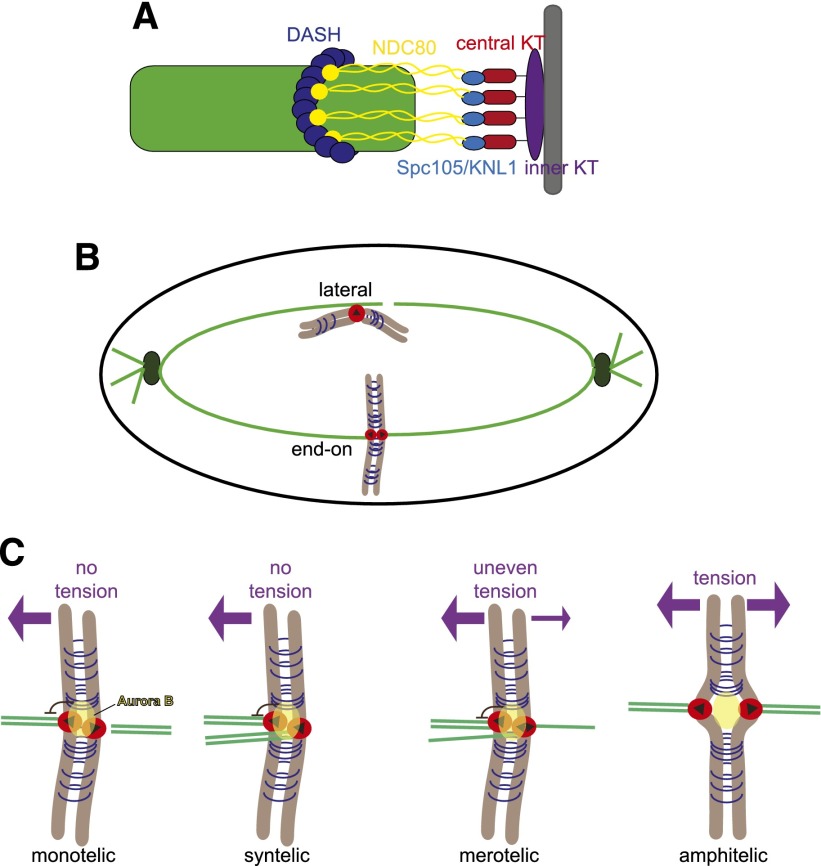
Kinetochore–microtubule interactions. (*A*) Diagram of the organization of the kinetochore. The inner kinetochore (purple) assembles on the centromeres of chromosomes (gray). The outer kinetochore (SPC105 [light blue] NDC80 [yellow], and DASH [blue ring]) forms the microtubule-binding interface. The central kinetochore (red) links the inner and outer subcomplexes of the kinetochore. The DASH complex ring is yeast-specific but is thought to be functionally analogous to the Ska1 complex in higher organisms. (*B*) Microtubules are first captured laterally by the kinetochore (red circles). These are then converted into stronger and more processive end-on attachments. The black triangles indicate kinetochore orientation. (*C*) Tension is generated when the pulling force of the microtubule is counteracted by cohesion (blue rings) holding sister chromatids together (see the text). Sister kinetochores are capable of capturing microtubules emanating from either spindle pole. Attachments that do not generate even tension allow Aurora B kinase (yellow) to sever kinetochore–microtubule attachments. Amphitelic attachments generate equal tension across sister kinetochores, thus removing Aurora B substrates from its reach.

The kinetochore can be thought of as comprising three parts: the inner kinetochore, which interacts with centromeric chromatin; the outer kinetochore, which directly interacts with spindle microtubules; and the central kinetochore, which connects the two (Fig. 2A; for excellent recent reviews of kinetochore structure, see Biggins 2013; Cheeseman 2014). Kinetochore proteins tend to be well conserved in different species, with some exceptions mostly in the inner kinetochore components (see Westermann and Schleiffer 2013 for a summary of homologs). The microtubule-binding elements of the outer kinetochore capture microtubules by chance: First, they associate with the microtubule lateral surface, which provides a larger contact surface compared with microtubule tips (Fig. 2B; Hayden et al. 1990; Rieder and Alexander 1990; Tanaka et al. 2005; Franco et al. 2007; Magidson et al. 2011). These initial lateral attachments are aided by the nucleation of additional microtubules at the kinetochore, which become integrated into the spindle (Kitamura et al. 2009). Additionally, in vertebrate cells, chromosomes modulate the local concentration of Ran-GTP to facilitate microtubule capture by kinetochores (Caudron et al. 2005; Kaláb et al. 2006). Lateral attachments are subsequently converted into the stronger and more processive end-on attachments. The kinetochore also directly modulates microtubule dynamics. Indeed, the recombinant Ndc80 complex favors rescue (the transition from microtubule shortening to growth) by directly stabilizing the tips of disassembling microtubules (Akiyoshi et al. 2010; Umbreit et al. 2012). Thus, the kinetochore both controls and harnesses the force generated by microtubules to direct chromosome segregation.

How does the kinetochore use the chemical energy of microtubule depolymerization to power chromosome movement? Careful tracking of chromosome movement and microtubule dynamics showed that disassembly of tubulin subunits from kinetochore-bound microtubule plus ends was associated with poleward chromosome movement (Gorbsky et al. 1987; Inoué and Salmon 1995). This suggested that kinetochores could move chromosomes toward the pole as they maintain their attachment to the disassembling plus end. Two nonmutually exclusive models have attempted to explain the mechanism by which kinetochores maintain the attachment with the disassembling tip to provide directional movement: the biased diffusion model and the conformational wave model (Fig. 1D; Asbury et al. 2011). In the first model, the kinetochore forms multiple additive and mobile interactions with microtubules (Hill 1985). Diffusion that increases the contacts with the microtubule favors attachment, thereby providing a biased direction. Lending support to this model, microtubule-binding elements are present at multiple copies in the kinetochore (Joglekar et al. 2006, 2008; Johnston et al. 2010), making kinetochores able to form multivalent attachments to microtubules, as indeed shown by electron microscopy (EM) studies of the budding yeast kinetochore (Gonen et al. 2012). Furthermore, recombinant Ndc80 and Dam1 complexes diffuse rapidly along the microtubule lattice (Westermann et al. 2005, 2006; Powers et al. 2009; Alushin et al. 2010). The alternate, conformational wave model postulates that as the microtubule protofilaments bend outward during depolymerization, they push on the kinetochore, pulling it along the microtubule (Koshland et al. 1988). The conformational wave model relies on a structure that would serve as a hook on which bending microtubules could push during disassembly. A microtubule-encircling ring has been proposed to be a possible mediator. In support of this model, the Dam1 complex in budding yeast forms a ring with 16-fold symmetry around microtubules in vitro (Miranda et al. 2005; Westermann et al. 2005), and EM studies show that budding yeast kinetochore rings often encircle microtubules (Gonen et al. 2012). However, in vitro studies have shown that the Dam1 complex is capable of tracking disassembling microtubules even in the absence of the ring structure (Gestaut et al. 2008; Grishchuk et al. 2008). Importantly, a purely conformational wave mechanism would predict that kinetochores would detach more quickly during assembly, when curling protofilaments are much less prominent (Mandelkow and Mandelkow 1985). However, single-molecule studies suggest that kinetochores actually detach more quickly during disassembly (Akiyoshi et al. 2010). It is likely that mechanisms and features proposed by both models contribute to the load-bearing attachments of kinetochores. Indeed, using the deformation of moving kinetochores as a readout of forces exerted on them, it was found that both active force generation within kinetochores and passive frictional interactions with microtubules contribute to these persistent attachments (Dumont et al. 2012).

### Holding sister DNA molecules together (cohesion)

Microtubules and kinetochores could, in principle, move chromosomes in any direction. Directionality in chromosome movement requires that sister chromatids be physically linked to provide an opposing force to that of the microtubules. Cohesin is the protein complex that achieves this by entrapping sister chromatids in a ring (for a recent review, see Marston 2014). Condensins, which are related to cohesins and also form a ring, give chromosomes their compact rod-shaped structure that allows their capture and movement during chromosome segregation (for review, see Hirano 2012). The pericentromere, the chromosomal region surrounding the centromere, is the region that experiences the highest levels of force, as evidenced by the separation of sister centromeres, but not arms, during metaphase in yeast (Goshima and Yanagida 2000; He et al. 2000; Tanaka et al. 2000). Both cohesin and condensin are highly enriched at the pericentromere (Blat and Kleckner 1999; Megee et al. 1999; Tanaka et al. 1999; Glynn et al. 2004; Kiburz et al. 2005; D’Ambrosio et al. 2008; Verzijlbergen et al. 2014) and are crucial to the architecture of pericentromeric chromatin in both yeast (Yong-Gonzalez et al. 2007; Ng et al. 2009; Stephens et al. 2013) and mammals (Ribeiro et al. 2009). Cohesin and condensin organize pericentromeric chromatin into a spring: Condensin compacts chromatin along the spindle axis, whereas cohesin localizes around the spindle axis and prevents the chromatin from spreading out radially (Stephens et al. 2011). This spatial confinement provides pericentromeric chromatin with the necessary rigidity to counterbalance spindle forces, allowing it to stretch, rather than break, under the force of the spindle. Furthermore, pericentromeres have been proposed to be cross-linked together, a feature that would allow a more efficient tension-based stabilization of multiple attachment sites (Stephens et al. 2013).

### Moving chromosomes in the right direction (orienting kinetochores)

As described above, kinetochores capture microtubules in an essentially stochastic way. However, faithful chromosome segregation requires that sister kinetochores attach to microtubules emanating from opposite spindle poles; this is termed amphitelic attachment, and the sister kinetochores are said to be bioriented (Fig. 2B). Sister kinetochores can also attach to microtubules from the same pole (syntelic attachments). Additionally, a single kinetochore can attach to microtubules from opposite poles, giving rise to merotelic attachments (Fig. 2B). Since both syntelic and merotelic attachments are not compatible with accurate chromosome segregation during mitosis, what mechanisms are in place to ensure that correct attachments are made? Tension lies at the heart of these mechanisms. The fundamental importance of tension was first made evident by elegant micromanipulation experiments with insect cells that showed that tension is used as a readout of accuracy (Li and Nicklas 1995; Nicklas et al. 1995; Nicklas 1997). When sister kinetochores are bioriented, the pulling force of spindle microtubules is counteracted by the cohesin linkages between sister chromatids, generating tension between sister kinetochores. This is the only mode of attachment that will exert equal force on each sister kinetochore, thereby producing even tension across them. Artificially applying tension on kinetochores both stabilizes and increases the number of microtubule–kinetochore attachments (Nicklas et al. 1995; King and Nicklas 2000). Tension stabilizes bipolar attachments by both direct (mechanical) and indirect (chemical) means. Kinetochores bind strongly to growing microtubules and weakly to shrinking microtubules. Tension suppresses microtubule disassembly, thus favoring the strongly bound state (Akiyoshi et al. 2010). In the indirect regulation, the pulling apart of sister centromeres removes kinetochores from the field of action of the kinase Aurora B, which continuously phosphorylates kinetochore components within its reach to disrupt kinetochore–microtubule attachments (Biggins et al. 1999; Tanaka et al. 2002; Liu et al. 2009; Welburn et al. 2010). Aurora B plays a crucial role in ensuring correct chromosome–microtubule attachments by releasing kinetochores in two ways: It weakens the attachment of the outer kinetochore proteins to the microtubule and directly destabilizes the kinetochore-attached microtubule tip (Lampson et al. 2004; Sarangapani et al. 2013).

Finally, in mitotic chromosomes, sister kinetochores are arranged in a way that favors biorientation. Indeed, sister kinetochores are thought to assume a back-to-back geometry, which favors their capture of microtubules from opposite poles (Figs. 2C, 4C [below]). Data from budding yeast point to the cohesin- and condensin-dependent pericentromere architecture producing an intrinsic bias of sister kinetochores toward biorientation (Indjeian and Murray 2007; Ng et al. 2009; Peplowska et al. 2014; Verzijlbergen et al. 2014).

Thus, the correct attachment of kinetochores to microtubules depends on both chromosome architecture (dictated by cohesin and condensin) and the generation of tension. Meiosis, the specialized cell division that gives rise to haploid gametes from a diploid progenitor cell, is guided by the same basic principles but with certain modifications to allow for a different chromosome segregation pattern.

## Specialization of the chromosome segregation machinery for meiosis

**Figure 3.**
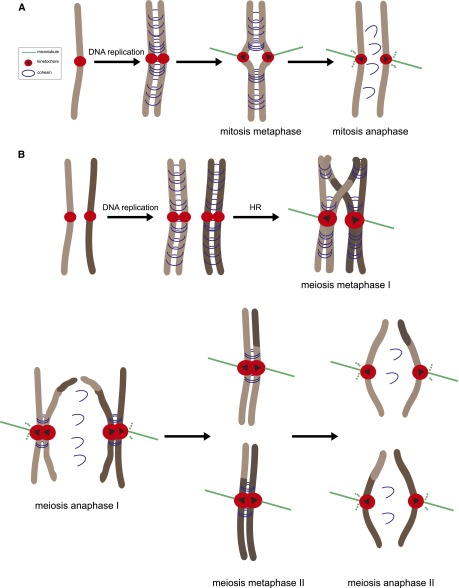
The chromosome segregation program in mitosis (*A*) and meiosis (*B*). During DNA replication, cohesin rings (blue) topologically entrap sister DNA molecules to give rise to sister chromatid cohesion. (*A*) In mitotic metaphase, sister kinetochores (red circles) are bioriented (black triangles): They attach to microtubules (green) emanating from opposite spindle poles. In anaphase, cohesin is cleaved, allowing sister chromatids to separate. (*B*) In meiotic prophase I, homologous recombination (HR) allows homologous chromosomes to be physically linked via chiasmata. In meiotic metaphase I, sister kinetochores are thought to fuse so as to present as a single microtubule-binding interface. In anaphase I, centromere-distal cohesin is cleaved, allowing homologous chromosomes to separate. Centromere cohesin, however, is protected. In meiosis II, much like in mitosis, sister kinetochores biorient in metaphase II, and the cleavage of centromere cohesin in anaphase II allows sister chromatids to segregate.

In meiosis, two rounds of segregation follow a single round of replication (for a review, see Marston and Amon 2004). In the first meiotic division (meiosis I), homologous chromosomes segregate away from each other, and sister chromatids comigrate. The first meiotic division is often called “reductional,” as it is this segregation event that results in the reduction in ploidy. In the second meiotic division (meiosis II), much like in mitosis, sister chromatids segregate (Fig. 3). Meiosis follows the same principles of chromosome segregation as mitosis; however, three important modifications underpin the first meiotic division: (1) Homologous chromosomes are physically linked together, usually by chiasmata, the products of homologous recombination. (2) Sister kinetochores attach to microtubules emanating from the same spindle pole (they are mono-oriented). (3) Cohesion is lost in a step-wise manner: Cohesion is lost on chromosome arms during the first division but is protected at the pericentromere (Fig. 3B). Importantly, these modifications are specified by properties of the chromosome rather than the spindle (Paliulis and Nicklas 2000). Nevertheless, the spindle does show meiosis-specific features in some organisms (Yi et al. 2013). It is likely that these adaptations are important for other developmental aspects of meiosis rather than for dictating the specialized chromosome segregation pattern (see below).

### Linking homologs

The biorientation of sister chromatids in mitosis relies on the tension created only by correct attachments: The pulling force of microtubules is counteracted by the physical linkages of cohesin between sister chromatids. In the first meiotic division, however, it is homologous chromosomes that must segregate away from each other. Meiosis relies on the same tension-based mechanisms for ensuring correct attachment of chromosomes to the spindle.

To enable these mechanisms to ensure biorientation of homologous chromosomes in meiosis I, physical linkages that can counteract spindle tension are generated between homologs by homologous recombination. Several excellent reviews (Baudat et al. 2013; Borde and de Massy 2013; de Massy 2013) have summarized recent strides in our understanding of homologous recombination in meiosis and its regulation. In many organisms, recombination is preceded by homologous chromosomes pairing along their lengths; this pairing is stabilized by synapsing through the assembly of a proteinaceous structure known as the synaptonemal complex (SC) (for reviews, see Zickler and Kleckner 1999; Bhalla and Dernburg 2008). Recombination starts in this context with the action of the endonuclease Spo11, which introduces deliberate and stochastic double-strand breaks along the chromosome (Keeney et al. 1997, 1999; Romanienko and Camerini-Otero 1999). A subset of these breaks is repaired using the nonsister homologous chromosome, creating physical links between homologs called chiasmata. The resulting homologous chromosome pair, called a bivalent, can now orient on the spindle, with interhomolog chiasmata resisting the pulling forces of the spindle. A single chiasma is sufficient to support the tension that is required for the accuracy of chromosome segregation (Hillers and Villeneuve 2003). It is at present unclear how the counterbalancing resistance provided by centromere-distal chiasmata is transmitted to kinetochores on the centromere. Whether meiotic chromosomes possess spring-like behavior at the point of tension, as observed for pericentromeres in mitosis, and whether increased structural rigidity of chromosome arms is required to allow force transduction along them remain questions for the future.

Once connections between homologs are made, homologous chromosomes need to attach to opposite poles so that they can segregate away from each other in anaphase I. In a manner similar to biorientation of sister chromatids in mitosis, tension and the action of Aurora B kinase play critical roles in achieving correct biorientation of homologs (Monje-Casas et al. 2007; Sakuno et al. 2011; Meyer et al. 2013).

### Protecting linkages between sister chromatids during meiosis I

Once homologs are bioriented, the links between them need to be severed to allow the poleward movement of chromosomes. In budding yeast, phosphorylation of the Rec8 cohesin subunit targets it for cleavage by separase (Brar et al. 2006; Ishiguro et al. 2010; Katis et al. 2010; Attner et al. 2013). Cohesin cleavage on chromosome arms resolves chiasmata, thereby allowing homologous chromosomes to segregate (Buonomo et al. 2000; Kudo et al. 2006). Cohesin in centromeric regions, however, must be protected from separase activity during meiosis I in order to ensure faithful segregation of sister chromatids in the second meiotic division. Genome-wide screens in budding yeast and fission yeast identified the shugoshin proteins, distant relatives of the fruit fly Mei-S332 protein, as essential factors protecting centromere cohesin at the end of the first division (Kerrebrock et al. 1992; Kitajima et al. 2004; Marston et al. 2004; Rabitsch et al. 2004). Shugoshin recruits the protein phosphatase PP2A to the pericentromere, which dephosphorylates Rec8, thereby rendering it refractory to separase cleavage (Kitajima et al. 2006; Riedel et al. 2006; Ishiguro et al. 2010; Katis et al. 2010). Residual pericentromeric cohesin provides the resistance to spindle forces during meiosis II, where sister kinetochores are bioriented in preparation for the mitosis-like segregation of sister chromatids to opposite poles.

### Cosegregation of sister chromatids during meiosis I

The comigration of sister chromatids in the first division requires that sister kinetochores be mono-oriented; i.e., attach to microtubules that emanate from the same spindle pole. The different arrangement of kinetochores in meiosis compared with mitosis was first reported from EM work in *Drosophila melanogaster* spermatocytes (Goldstein 1981), where sister kinetochores were shown to be very closely associated. This suggested a mechanism for mono-orientation whereby sister kinetochores “fuse”; i.e., they create a single microtubule-binding interface. Recent support for this model came from fluorescence microscopy studies in maize meiocytes. Li and Dawe (2009) found that the MIS12 and NDC80 kinetochore components span across the sister centromeres to form a direct cross-linking bridge between the sister kinetochores. Knockdown of MIS12 by RNAi weakened this link and caused a third of sister chromatids to segregate away from each other (Li and Dawe 2009). While these studies have been critical for understanding higher eukaryote sister mono-orientation, the bulk of the molecular insights into mono-orientation mechanisms has come from studies in budding and fission yeasts.

#### Budding yeast and sister kinetochore fusion

**Figure 4.**
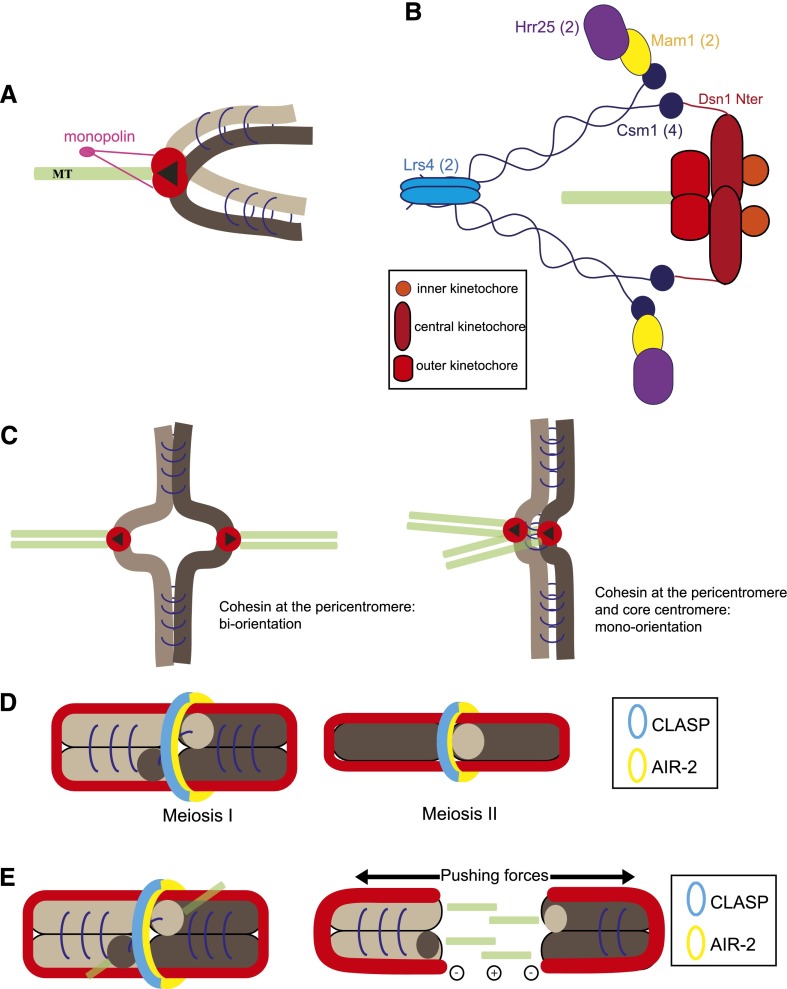
Mono-orientation of sister kinetochores in meiosis I. (*A*) Monopolin (pink) cross-links sister kinetochores to create a single microtubule-binding interface. (*B*) Diagram of the organization of the monopolin complex. Csm1 (dark blue) and Lrs4 (light blue) form a V-shaped complex, with the Csm1 globular heads spaced at 10 nm apart. Csm1 is thought to interact directly with the N terminus of Dsn1 (red) via Csm1’s globular head. The other globular head interacts with Mam1 (yellow), which in turn recruits a copy of casein kinase (Hrr25, purple). The copy number of each protein in the complex is indicated in brackets. (*C*) In fission yeast, sister kinetochore orientation is determined by centromeric cohesion: When there is no cohesion at the core centromere, sister kinetochore biorient (*left*); cohesion at the core centromere allows for mono-orientation in meiosis I (*right*). (*D*) In *C. elegans*, a kinetochore sheath forms around the bivalent in meiosis I (*left*) or sister chromatids in meiosis II (*right*). Aurora B kinase (AIR-2, yellow) forms a ring around the mid-bivalent (meiosis I) or sister chromatid interface (meiosis II). AIR-2 is also thought to mark the site for CLASP-dependent microtubule growth that pushes the dividing chromosomes apart in a kinetochore-independent manner. (*E*) Kinetochores are not responsible for chromosome motion in *C. elegans* oocytes. Instead, the microtubule-stabilizing protein CLASP promotes microtubule polymerization between chromosomes. This microtubule growth could generate the force required for the segregation of chromosomes.

The first suggestion for a sister kinetochore fusion model in budding yeast meiosis was inspired by EM studies of the meiosis I spindle, which showed that the number of kinetochore microtubules was not sufficient for each sister kinetochore to be attached independently to the spindle (Winey et al. 2005). Many studies have since supported the view that in budding yeast, mono-orientation is achieved by the rearrangement of sister kinetochores to create a single microtubule-binding unit (Fig. 4A). Crucial to this rearrangement is monopolin, a four-protein complex identified by functional genomics and proteomics (Tóth et al. 2000; Rabitsch et al. 2003; Petronczki et al. 2006). Monopolin consists of Mam1, a protein expressed exclusively during the first meiotic division; casein kinase Hrr25; and the proteins Csm1 and Lrs4 (Fig. 4B). Monopolin associates with kinetochores from late prophase I (when the expression of the *MAM1* gene is induced) until the end of the first meiotic division (Tóth et al. 2000). At least two additional kinases play important roles in the function of monopolin: polo-like kinase Cdc5 and the Dbf4-dependent kinase (DDK). Cdc5 releases Csm1 and Lrs4 from the nucleolus, where they normally reside (Clyne et al. 2003; Lee and Amon 2003; Rabitsch et al. 2003); subsequently, Cdc5 and DDK together act to phosphorylate Lrs4 (Matos et al. 2008). Whether these are the only critical functions that these kinases play in mono-orientation is not yet known. The stable association of monopolin with kinetochores also requires Spo13, a meiosis I-specific protein that regulates many aspects of budding yeast meiosis in poorly understood ways (Shonn et al. 2002; Katis et al. 2004; Lee et al. 2004). Recently, it was shown that mono-orientation in budding yeast also depends on the temporal regulation of kinetochore–microtubule attachments (Miller et al. 2012). Kinetochore–microtubule attachments are abolished in prophase of the first meiotic division because Ndc80, which provides the main microtubule-binding activity of the kinetochore, is destabilized in an Ipl1-dependent manner (Miller et al. 2012; Kim et al. 2013). If kinetochores are induced to engage with microtubules prematurely, mono-orientation is not established (Miller et al. 2012). It seems likely that monopolin cannot bind to kinetochores that are already associated with microtubules.

Monopolin is recruited to kinetochores by binding to the N terminus of Dsn1 (Mis13 in other organisms), a component of the MIND complex of the central kinetochore (Fig. 4B; Sarkar et al. 2013; Sarangapani et al. 2014). Notably, this could also act as the point at which sister kinetochores are cross-linked into a single functional unit. The crystal structure of the Csm1:Lrs4 complex was the first to provide strong evidence for direct cross-linking of sister kinetochores (Corbett et al. 2010; Corbett and Harrison 2012). Csm1:Lrs4 form a V-shaped complex, with each of the globular “heads” containing a dimer of Csm1 C-terminal domains (Fig. 4B). This suggested that each “head” could bind to a different sister kinetochore to bring about cross-linking. This has been further supported by data showing that N-terminally truncated Dsn1 dominantly prevents sister kinetochore mono-orientation in meiosis I when produced in the presence of wild-type Dsn1 (Sarkar et al. 2013). This points to the N terminus of Dsn1 as a potential cross-linking site for kinetochores (Fig. 4B).

More recently, single-molecule techniques have been used to directly assess the role of monopolin in fusing sister kinetochores. Kinetochores purified from meiosis I withstand more force and have more microtubule-binding elements than mitotic kinetochores (Sarangapani et al. 2014). This is unlikely to be solely due to monopolin-induced rearrangements in the architecture of individual kinetochores: DNA replication and thus the presence of a sister kinetochore are required for this increase in strength (Sarangapani et al. 2014). Importantly, monopolin was able to increase the strength of mitotic kinetochores in vitro in a manner that was independent of casein kinase activity (Sarangapani et al. 2014). The latter highlights our lack of understanding of the role of casein kinase in promoting mono-orientation. The kinase activity of Hrr25 is required for mono-orientation but not for the recruitment of monopolin (Petronczki et al. 2006). Intriguingly, Hrr25 phosphorylates Mam1 in a manner that destabilizes the monopolin complex (Corbett and Harrison 2012). This suggests that Hrr25 might phosphorylate kinetochore components to either achieve mono-orientation or ensure that the correct sisters are fused. The precise role played by Mam1 is also unclear. Mam1 is unlikely to act simply as a recruitment factor, since both Csm1 and Lrs4 are recruited to kinetochores in mitotic anaphase, when Mam1 is not expressed (Akiyoshi et al. 2009; Brito et al. 2010). Furthermore, Mam1 binds to Csm1 to occlude one of its kinetochore-binding interfaces (Corbett and Harrison 2012). In vitro and structural work will be required to elucidate the precise nature of sister kinetochore fusion and the role played therein by each component of monopolin.

#### Fission yeast and the role of cohesion in mono-orientation

In fission yeast, mono-orientation has been shown to be driven by modifications in centromere cohesion (Sakuno et al. 2009). Fission yeast centromeres are composed of heterochromatic outer repeats and a central core domain containing CENP-A nucleosomes. In mitosis, cohesin is highly enriched at the outer repeats flanking the central domain of the centromere, which is depleted for cohesin; this is thought to favor a back-to-back configuration of sister kinetochores, which in turn promotes their biorientation (Fig. 4C, left panel). In meiosis, however, the meiosis-specific cohesin subunit Rec8 is enriched also at the central core of the centromere, which is thought to favor a side-by-side arrangement of sister kinetochores to allow their efficient mono-orientation (Fig. 4C, right panel; Watanabe and Nurse 1999; Sakuno et al. 2009). The meiosis-specific factor Moa1 is recruited to the kinetochore via the inner centromere protein CENP-C and promotes cohesion at the core centromere (Yokobayashi and Watanabe 2005; Tanaka et al. 2009). Rec8 disappearance from the core centromere before prophase II would allow for the biorientation of sister kinetochores in meiosis II. Here it is important to note that Sgo1, which protects centromere cohesin from being cleaved in anaphase I, localizes only at the pericentromere and not at the core centromere (Sakuno et al. 2009). Lending further support to the importance of geometry for mono-orientation, an artificial tether between the central domains of sister centromeres restored mono-orientation in meiosis I in the absence of Rec8 (Sakuno et al. 2009). However, centromeric tethers induced in mitosis led to a modest increase in cosegregation of sister chromatids (Sakuno et al. 2009). In contrast, ectopic localization of monopolin to the mitotic kinetochore in budding yeast leads to up to 50% mono-orientation of sister kinetochores (Monje-Casas et al. 2007; Miller et al. 2012). Thus, other factors are very likely to be required for mono-orientation in fission yeast.

Rec8 has been shown to play a role in many other organisms, including mice (Parra et al. 2004), *Arabidopsis thaliana* (Chelysheva et al. 2005), and *Caenorhabditis elegans* (Severson et al. 2009). This is in contrast to budding yeast, where Rec8 does not seem to be required for establishing mono-orientation of sister kinetochores (Tóth et al. 2000; Monje-Casas et al. 2007). This could stem from the very different nature of the budding yeast centromere and its kinetochore–microtubule attachments. Budding yeast has simple point centromeres of 125 base pairs (bp) with no flanking heterochromatin, which would not allow for accurate differential organizations of centromere cohesion. Furthermore, in budding yeast, one kinetochore (or a pair of sister kinetochores in meiosis I) attaches to a single microtubule (Winey et al. 2005). In fission yeast, on the other hand, multiple microtubules (two to three) bind a single kinetochore. This might make sister kinetochore fusion a much more efficient mechanism of mono-orientation in budding yeast. The ability to clamp together microtubule-binding elements suggested that monopolin might have a conserved role in other organisms to prevent merotely (Rabitsch et al. 2003). Indeed, Csm1 and Lrs4 homologs in fission yeast (Pcs1 and Mde4) are required for suppressing merotelic attachments in mitosis (Gregan et al. 2007) even though they are dispensable for sister kinetochore mono-orientation (Rabitsch et al. 2003). Importantly, unlike budding yeast, where monopolin seems to clamp microtubule-binding sites directly (Corbett et al. 2010), fission yeast Pcs1 and Mde4 have been shown to prevent merotely by recruiting condensin to centromeres (Tada et al. 2011).

Last, the finding that meiosis-specific cohesins are necessary for sister kinetochore mono-orientation in many organisms but are not sufficient (at least in fission yeast) suggests that additional factors are required. This raises the possibility that undiscovered factors that are functionally analogous to budding yeast monopolin cross-link kinetochores in these organisms. Potentially, meiosis-specific cohesins in the centromeric region may be required to place sister kinetochores in sufficient proximity to allow their cross-linking. This would be envisaged to be especially important in organisms with large centromeres that contact multiple microtubules, while in organisms with point centromeres, monopolin may in itself be sufficient.

### Other unusual features of meiosis in some organisms

#### Sister kinetochore mono-orientation in holocentric chromosomes

So far, our discussion has been focused on chromosomes where the kinetochore assembles on a single locus; these are called monocentric chromosomes because they have one centromere. In some organisms, however, kinetochores assemble along the entire length of the chromosome; these are termed holocentric chromosomes. Organisms with holocentric chromosomes include the nematode worm *C. elegans* and also some insects, arachnids, and several plant species (Albertson and Thomson 1993). One important consequence of holocentric chromosomes is that microtubules can attach at several points along them, so the pulling forces of the spindle are not exerted at a single point. Additionally, this imposes certain modifications for meiotic chromosome segregation. In meiosis I, homologous chromosomes are linked by chiasmata to form a cruciform bivalent (Fig. 3B). Because kinetochores assemble alongside the length of the chromosome, holocentric bivalents could have microtubule-binding surfaces facing in all directions. To resolve this, kinetochores assemble to form a sheath that encapsulates bivalents (Fig. 4D). Interestingly, and in stark contrast to mitosis, the cup-like assembly of kinetochores on holocentric chromosomes in meiosis does not require CENP-A (Dumont et al. 2010). The meiosis-specific cohesin Rec8 plays a fundamental role in connecting the sister chromatids so that they can be encapsulated (Severson et al. 2009). The fact that Rec8 directs key aspects of chromosome segregation in organisms whose kinetochores are arranged in dramatically different ways points to an early adaptation of meiotic cohesin in establishing the meiotic chromosome segregation program.

#### Segregation of homologs without chiasmata

Despite the importance of homologous recombination in the segregation of chromosomes in meiosis I, chromosomes can segregate accurately in the absence of visible chiasmata. In budding yeast, for instance, a single nonexchange (achiasmatic) chromosome segregates faithfully in 90% of meioses (Dawson et al. 1986; Mann and Davis 1986; Guacci and Kaback 1991), and in many organisms, sex chromosomes segregate without any recombination at all (for review, see Wolf 1994). Achiasmatic chromosomes often form physical associations by alternative mechanisms, which allow the generation of tension when they biorient on the meiosis I spindle. In the female silk moth, *Bombyx mori*, the SC remains associated with the bivalents until anaphase I (Rasmussen 1977), presumably functioning as a substitute for chiasmata. In some mammals, SC proteins (e.g., SYCP3) form links between sex chromosomes (Page et al. 2006; de la Fuente et al. 2007). This is reminiscent of the role the budding yeast SC component Zip1 plays to pair centromeres in early meiotic prophase, which is thought to promote the biorientation of homologs (Tsubouchi and Roeder 2005; Gladstone et al. 2009; Newnham et al. 2010). Together, these data point to an ancient role for SC proteins in maintaining association of homologous achiasmatic chromosomes. In fruit fly oocytes, heterochromatic threads provide a substitute link between the achiasmatic fourth chromosomes (Dernburg et al. 1996; Karpen et al. 1996). In fruit fly male meiosis, however, there is no SC, and heterochromatic regions only play a role in a subset of chromosomes (Tsai et al. 2011). Here, the necessary connection is provided by the nucleolar rDNA regions in a manner dependent on male meiosis-specific proteins SNM and MNM (Thomas et al. 2005).

#### Poleward microtubule flux

We have seen how the major force for chromosome motion is provided by the disassembly of microtubules at the kinetochore-bound plus end. However, microtubules also disassemble at their minus end on the spindle pole in a process termed poleward microtubule flux (Fig. 1E). Microtubule flux has been shown to play important roles in chromosome congression in metaphase (for review, see Ganem and Compton 2006). However, it can also generate force. Indeed, microtubule flux may be particularly important in meiosis to support chromosome motion. Microtubule-marking experiments have allowed the rate of poleward microtubule flux to be measured. By comparing the rate of flux with the velocity of chromosome motion, one can estimate the contribution of poleward microtubule flux to chromosome movement. Strikingly, in many of the meiotic cells that have been studied, such as *Xenopus* egg extracts and insect spermatocytes, flux velocity meets or exceeds chromosome velocity (Desai et al. 1998; LaFountain et al. 2004), strongly suggesting that in these systems, poleward microtubule flux is likely to be the primary mechanism driving chromosome segregation. Conversely, in somatic cells that have been studied, such as PtK1, PtK2, LLC-PK1, Newt lung, and HeLa, poleward microtubule flux makes a small contribution to the chromosome movement (Ganem et al. 2005).

#### Chromosome segregation without kinetochores or centrosomes

In the “canonical” system of chromosome segregation depicted in Figure 1A, kinetochores capture microtubules emanating from the spindle pole, the centrosome, to power chromosome motion. However, in many organisms, including fruit flies, *C. elegans,* mice, and humans, female meiosis takes place in the absence of centrosomes. This could be an adaptation that avoids the formation of multipolar spindles once sperm carrying the centrosome enter after fertilization. In these acentrosomal divisions, the spindle is assembled from the self-organization of many alternative microtubule-organizing centers (Schuh and Ellenberg 2007), often including chromatin itself (Heald et al. 1996). A striking example of how acentrosomal meiosis redefines basic principles of chromosome segregation is provided by *C. elegans*. *C. elegans* oocytes do not rely on kinetochores to power the separation of chromosomes; kinetochores are simply required to orient chromosomes in the spindle (Dumont et al. 2010). Instead, microtubules nucleate between the separating bivalents, and it is this that drives the separation of chromosomes (Fig. 4E). The microtubule dynamics regulator protein CLASP as well as Aurora B kinase and the kinase BUB-1 form a ring at the midbivalent that nucleates microtubules. The directed growth of these bundles of microtubules pushes the homologs apart. Why might such a kinetochore-independent mechanism have arisen? Dumont and Desai (2012) suggested that it might be an adaptation of holocentric chromosomes: Aurora kinase associates with the site of crossover, thereby marking both the site for cohesin loss (Schvarzstein et al. 2010) and the site for microtubule growth that will drive segregation. This ensures that homologs, but not sister chromatids, segregate in the first division. Alternatively, or in addition, this may represent a more general mechanism used in acentrosomal division. DNA-coated beads separate in mouse oocytes in the absence of kinetochore function (Deng et al. 2009), suggesting that kinetochore-independent mechanisms may be in place to generate the force that powers chromosome segregation in acentrosomal meiosis of other organisms.

## Conclusion

Although a wealth of studies has provided detailed insights into how chromosomes are moved during mitosis, key questions remain about how microtubule-generated force is coupled to chromosomes by the remarkable molecular machine that is the kinetochore. During meiosis, adaptations to both kinetochores and the spindle alter the way that force is generated and used. What is the biological rationale underlying these modifications? How do they effect the specialized pattern of meiotic chromosome segregation at the molecular level? The biochemical reconstitution of many components of the cell division machinery coupled with high-resolution imaging of live cells will allow for a plethora of exciting questions to be answered.

## References

[B1] Akiyoshi B, Nelson CR, Ranish JA, Biggins S 2009 Quantitative proteomic analysis of purified yeast kinetochores identifies a PP1 regulatory subunit. Genes Dev 23: 2887–2899. 19948764 10.1101/gad.1865909 PMC2800092

[B2] Akiyoshi B, Sarangapani KK, Powers AF, Nelson CR, Reichow SL, Arellano-Santoyo H, Gonen T, Ranish JA, Asbury CL, Biggins S 2010 Tension directly stabilizes reconstituted kinetochore–microtubule attachments. Nature 468: 576–579. 21107429 10.1038/nature09594 PMC3108429

[B3] Albertson D, Thomson JN 1993 Segregation of holocentric chromosomes at meiosis in the nematode, *Caenorhabditis elegans*. Chromosome Res 1: 15–26. 8143084 10.1007/BF00710603

[B4] Alushin GM, Ramey VH, Pasqualato S, Ball DA, Grigorieff N, Musacchio A, Nogales E 2010 The Ndc80 kinetochore complex forms oligomeric arrays along microtubules. Nature 467: 805–810. 20944740 10.1038/nature09423 PMC2957311

[B5] Asbury CL, Tien JF, Davis TN 2011 Kinetochores’ gripping feat: conformational wave or biased diffusion? Trends Cell Biol 21: 38–46. 20951587 10.1016/j.tcb.2010.09.003 PMC3075839

[B6] Attner MA, Miller MP, Ee L-s, Elkin SK, Amon A 2013 Polo kinase Cdc5 is a central regulator of meiosis I. Proc Natl Acad Sci 110: 14278–14283. 23918381 10.1073/pnas.1311845110 PMC3761645

[B7] Baudat F, Imai Y, de Massy B 2013 Meiotic recombination in mammals: localization and regulation. Nat Rev Genet 14: 794–806. 24136506 10.1038/nrg3573

[B8] Bhalla N, Dernburg AF 2008 Prelude to a division. Annu Rev Cell Dev Biol 24: 397–424. 18597662 10.1146/annurev.cellbio.23.090506.123245 PMC4435778

[B9] Biggins S 2013 The composition, functions, and regulation of the budding yeast kinetochore. Genetics 194: 817–846. 23908374 10.1534/genetics.112.145276 PMC3730914

[B10] Biggins S, Severin FF, Bhalla N, Sassoon I, Hyman AA, Murray AW 1999 The conserved protein kinase Ipl1 regulates microtubule binding to kinetochores in budding yeast. Genes Dev 13: 532–544. 10072382 10.1101/gad.13.5.532 PMC316509

[B11] Blat Y, Kleckner N 1999 Cohesins bind to preferential sites along yeast chromosome III, with differential regulation along arms versus the centric region. Cell 98: 249–259. 10428036 10.1016/s0092-8674(00)81019-3

[B12] Borde V, de Massy B 2013 Programmed induction of DNA double strand breaks during meiosis: setting up communication between DNA and the chromosome structure. Curr Opin Genet Dev 23: 147–155. 23313097 10.1016/j.gde.2012.12.002

[B13] Brar GA, Kiburz BM, Zhang Y, Kim J-E, White F, Amon A 2006 Rec8 phosphorylation and recombination promote the step-wise loss of cohesins in meiosis. Nature 441: 532–536. 16672979 10.1038/nature04794

[B14] Brito I, Monje-Casas F, Amon A 2010 The Lrs4–Csm1 monopolin complex associates with kinetochores during anaphase and is required for accurate chromosome segregation. Cell Cycle 9: 3611–3618. 20818155 10.4161/cc.9.17.12885 PMC3047622

[B15] Buonomo SBC, Clyne RK, Fuchs J, Loidl J, Uhlmann F, Nasmyth K 2000 Disjunction of homologous chromosomes in meiosis I depends on proteolytic cleavage of the meiotic cohesin Rec8 by Separin. Cell 103: 387–398. 11081626 10.1016/s0092-8674(00)00131-8

[B16] Caudron M, Bunt G, Bastiaens P, Karsenti E 2005 Spatial coordination of spindle assembly by chromosome-mediated signaling gradients. Science 309: 1373–1376. 16123300 10.1126/science.1115964

[B17] Cheeseman IM 2014 The kinetochore. Cold Spring Harb Perspect Biol 6: a015826. 24984773 10.1101/cshperspect.a015826 PMC4067989

[B18] Chelysheva L, Diallo S, Vezon D, Gendrot G, Vrielynck N, Belcram K, Rocques N, Márquez-Lema A, Bhatt AM, Horlow C, 2005 AtREC8 and AtSCC3 are essential to the monopolar orientation of the kinetochores during meiosis. J Cell Sci 118: 4621–4632. 16176934 10.1242/jcs.02583

[B19] Clyne RK, Katis VL, Jessop L, Benjamin KR, Herskowitz I, Lichten M, Nasmyth K 2003 Polo-like kinase Cdc5 promotes chiasmata formation and cosegregation of sister centromeres at meiosis I. Nat Cell Biol 5: 480–485. 12717442 10.1038/ncb977

[B20] Corbett KD, Harrison SC 2012 Molecular architecture of the yeast monopolin complex. Cell Reports 1: 583–589. 22813733 10.1016/j.celrep.2012.05.012 PMC3494995

[B21] Corbett KD, Yip CK, Ee L-S, Walz T, Amon A, Harrison SC 2010 The monopolin complex crosslinks kinetochore components to regulate chromosome–microtubule attachments. Cell 142: 556–567. 20723757 10.1016/j.cell.2010.07.017 PMC2955198

[B22] D’Ambrosio C, Schmidt CK, Katou Y, Kelly G, Itoh T, Shirahige K, Uhlmann F 2008 Identification of *cis*-acting sites for condensin loading onto budding yeast chromosomes. Genes Dev 22: 2215–2227. 18708580 10.1101/gad.1675708 PMC2518811

[B23] Dawson D, Murray A, Szostak J 1986 An alternative pathway for meiotic chromosome segregation in yeast. Science 234: 713–717. 3535068 10.1126/science.3535068

[B24] de la Fuente R, Parra MT, Viera A, Calvente A, Gómez R, Suja JÁ, Rufas JS, Page J 2007 Meiotic pairing and segregation of achiasmate sex chromosomes in eutherian mammals: the role of SYCP3 orotein. PLoS Genet 3: e198. 17983272 10.1371/journal.pgen.0030198 PMC2048527

[B25] de Massy B 2013 Initiation of meiotic recombination: how and where? Conservation and specificities among eukaryotes. Annu Rev Genet 47: 563–599. 24050176 10.1146/annurev-genet-110711-155423

[B26] Deng M, Gao J, Suraneni P, Li R 2009 Kinetochore-independent chromosome poleward movement during anaphase of meiosis II in mouse eggs. PLoS ONE 4: e5249. 19365562 10.1371/journal.pone.0005249 PMC2664963

[B27] Dernburg AF, Sedat JW, Hawley RS 1996 Direct evidence of a role for heterochromatin in meiotic chromosome segregation. Cell 86: 135–146. 8689681 10.1016/s0092-8674(00)80084-7

[B28] Desai A, Mitchison TJ 1997 Microtubule polymerization dynamics. Annu Rev Cell Dev Biol 13: 83–117. 9442869 10.1146/annurev.cellbio.13.1.83

[B29] Desai A, Maddox PS, Mitchison TJ, Salmon ED 1998 Anaphase A chromosome movement and poleward spindle microtubule flux occur at similar rates in *Xenopus* extract spindles. J Cell Biol 141: 703–713. 9566970 10.1083/jcb.141.3.703 PMC2132746

[B157] Dumont J, Desai A 2012 Acentrosomal spindle assembly and chromosome segregation during oocyte meiosis. Trends Cell Biol 22: 241–249. 22480579 10.1016/j.tcb.2012.02.007 PMC3348331

[B30] Dumont J, Oegema K, Desai A 2010 A kinetochore-independent mechanism drives anaphase chromosome separation during acentrosomal meiosis. Nat Cell Biol 12: 894–901. 20729837 10.1038/ncb2093 PMC3052858

[B31] Dumont S, Salmon ED, Mitchison TJ 2012 Deformations within moving kinetochores reveal different sites of active and passive force generation. Science 337: 355–358. 22722252 10.1126/science.1221886 PMC3672420

[B32] Endow S, Kang S, Satterwhite L, Rose M, Skeen V, Salmon E 1994 Yeast Kar3 is a minus-end microtubule motor protein that destabilizes microtubules preferentially at the minus ends. EMBO J 13: 2708. 7912193 10.1002/j.1460-2075.1994.tb06561.x PMC395145

[B33] Ferraro-Gideon J, Sheykhani R, Zhu Q, Duquette ML, Berns MW, Forer A 2013 Measurements of forces produced by the mitotic spindle using optical tweezers. Mol Biol Cell 24: 1375–1386. 23485565 10.1091/mbc.E12-12-0901 PMC3639049

[B34] Franco A, Meadows JC, Millar JBA 2007 The Dam1/DASH complex is required for the retrieval of unclustered kinetochores in fission yeast. J Cell Sci 120: 3345–3351. 17881496 10.1242/jcs.013698

[B35] Gaglio T, Saredi A, Bingham J, Hasbani M, Gill S 1996 Opposing motor activities are required for the organization of the mammalian mitotic spindle pole. J Cell Biol 135: 399. 8896597 10.1083/jcb.135.2.399 PMC2121053

[B36] Ganem N, Compton D 2006 Functional roles of poleward microtubule flux during mitosis. Cell Cycle 5: 481–485. 16552178 10.4161/cc.5.5.2519

[B37] Ganem NJ, Upton K, Compton DA 2005 Efficient mitosis in human cells lacking poleward microtubule flux. Curr Biol 15: 1827–1832. 16243029 10.1016/j.cub.2005.08.065

[B38] Gestaut DR, Graczyk B, Cooper J, Widlund PO, Zelter A, Wordeman L, Asbury CL, Davis TN 2008 Phosphoregulation and depolymerization-driven movement of the Dam1 complex do not require ring formation. Nat Cell Biol 10: 407–414. 18364702 10.1038/ncb1702 PMC2782782

[B39] Gladstone MN, Obeso D, Chuong H, Dawson DS 2009 The synaptonemal complex protein Zip1 promotes bi-orientation of centromeres at meiosis I. PLoS Genet 5: e1000771. 20011112 10.1371/journal.pgen.1000771 PMC2781170

[B40] Glynn EF, Megee PC, Yu H-G, Mistrot C, Unal E, Koshland DE, DeRisi JL, Gerton JL 2004 Genome-wide mapping of the cohesin complex in the yeast *Saccharomyces cerevisiae*. PLoS Biol 2: e259. 15309048 10.1371/journal.pbio.0020259 PMC490026

[B41] Goldstein L 1981 Kinetochore structure and its role in chromosome orientation during the first meiotic division in male *D. melanogaster*. Cell 25: 591–602. 6793236 10.1016/0092-8674(81)90167-7

[B42] Gonen S, Akiyoshi B, Iadanza MG, Shi D, Duggan N, Biggins S, Gonen T 2012 The structure of purified kinetochores reveals multiple microtubule-attachment sites. Nat Struct Mol Biol 19: 925–929. 22885327 10.1038/nsmb.2358 PMC3443262

[B43] Gorbsky GJ, Sammak PJ, Borisy GG 1987 Chromosomes move poleward in anaphase along stationary microtubules that coordinately disassemble from their kinetochore ends. J Cell Biol 104: 9–18. 3793763 10.1083/jcb.104.1.9 PMC2117032

[B44] Goshima G, Yanagida M 2000 Establishing biorientation occurs with precocious separation of the sister kinetochores, but not the arms, in the early spindle of budding yeast. Cell 100: 619–633. 10761928 10.1016/s0092-8674(00)80699-6

[B45] Gregan J, Riedel CG, Pidoux AL, Katou Y, Rumpf C, Schleiffer A, Kearsey SE, Shirahige K, Allshire RC, Nasmyth K 2007 The kinetochore proteins Pcs1 and Mde4 and heterochromatin are required to prevent merotelic orientation. Current Biol 17: 1190–1200. 10.1016/j.cub.2007.06.044 PMC1931489 17627824

[B46] Grishchuk EL, McIntosh JR 2006 Microtubule depolymerization can drive poleward chromosome motion in fission yeast. EMBO J 25: 4888–4896. 17036054 10.1038/sj.emboj.7601353 PMC1618090

[B47] Grishchuk EL, Molodtsov MI, Ataullakhanov FI, McIntosh JR 2005 Force production by disassembling microtubules. Nature 438: 384–388. 16292315 10.1038/nature04132

[B48] Grishchuk EL, Spiridonov IS, Volkov VA, Efremov A, Westermann S, Drubin D, Barnes G, Ataullakhanov FI, McIntosh JR 2008 Different assemblies of the DAM1 complex follow shortening microtubules by distinct mechanisms. Proc Natl Acad Sci 105: 6918–6923. 18460602 10.1073/pnas.0801811105 PMC2383981

[B49] Guacci V, Kaback DB 1991 Distributive disjunction of authentic chromosomes in *Saccharomyces cerevisiae*. Genetics 127: 475–488. 2016050 10.1093/genetics/127.3.475 PMC1204375

[B50] Hayden JH, Bowser SS, Rieder CL 1990 Kinetochores capture astral microtubules during chromosome attachment to the mitotic spindle: direct visualization in live newt lung cells. J Cell Biol 111: 1039–1045. 2391359 10.1083/jcb.111.3.1039 PMC2116290

[B51] He X, Asthana S, Sorger PK 2000 Transient sister chromatid separation and elastic deformation of chromosomes during mitosis in budding yeast. Cell 101: 763–775. 10892747 10.1016/s0092-8674(00)80888-0

[B52] Heald R, Tournebize R, Blank T, Sandaltzopoulos R, Becker P 1996 Self-organization of microtubules into bipolar spindles around artificial chromosomes in *Xenopus* egg extracts. Nature 382: 420. 8684481 10.1038/382420a0

[B53] Hill TL 1985 Theoretical problems related to the attachment of microtubules to kinetochores. Proc Natl Acad Sci 82: 4404–4408. 3859869 10.1073/pnas.82.13.4404 PMC391109

[B54] Hillers KJ, Villeneuve AM 2003 Chromosome-wide control of meiotic crossing over in *C. elegans*. Curr Biol 13: 1641–1647. 13678597 10.1016/j.cub.2003.08.026

[B55] Hirano T 2012 Condensins: universal organizers of chromosomes with diverse functions. Genes Dev 26: 1659–1678. 22855829 10.1101/gad.194746.112 PMC3418584

[B56] Indjeian VB, Murray AW 2007 Budding yeast mitotic chromosomes have an intrinsic bias to biorient on the spindle. Curr Biol 17: 1837–1846. 17980598 10.1016/j.cub.2007.09.056

[B57] Inoué S, Salmon E 1995 Force generation by microtubule assembly/disassembly in mitosis and related movements. Mol Biol Cell 6: 1619. 8590794 10.1091/mbc.6.12.1619 PMC301321

[B58] Ishiguro T, Tanaka K, Sakuno T, Watanabe Y 2010 Shugoshin-PP2A counteracts casein-kinase-1-dependent cleavage of Rec8 by separase. Nat Cell Biol 12: 500–506. 20383139 10.1038/ncb2052

[B59] Joglekar AP, Bouck DC, Molk JN, Bloom KS, Salmon ED 2006 Molecular architecture of a kinetochore–microtubule attachment site. Nat Cell Biol 8: 581–585. 16715078 10.1038/ncb1414 PMC2867088

[B60] Joglekar AP, Bouck D, Finley K, Liu X, Wan Y, Berman J, He X, Salmon ED, Bloom KS 2008 Molecular architecture of the kinetochore–microtubule attachment site is conserved between point and regional centromeres. J Cell Biol 181: 587–594. 18474626 10.1083/jcb.200803027 PMC2386099

[B61] Johnston K, Joglekar A, Hori T, Suzuki A, Fukagawa T, Salmon ED 2010 Vertebrate kinetochore protein architecture: protein copy number. J Cell Biol 189: 937–943. 20548100 10.1083/jcb.200912022 PMC2886349

[B62] Kaláb P, Pralle A, Isacoff EY, Heald R, Weis K 2006 Analysis of a RanGTP-regulated gradient in mitotic somatic cells. Nature 440: 697–701. 16572176 10.1038/nature04589

[B63] Kapoor TM, Lampson MA, Hergert P, Cameron L, Cimini D, Salmon ED, McEwen BF, Khodjakov A 2006 Chromosomes can congress to the metaphase plate before biorientation. Science 311: 388–391. 16424343 10.1126/science.1122142 PMC4768465

[B64] Karpen GH, Le M-H, Le H 1996 Centric heterochromatin and the efficiency of achiasmate disjunction in *Drosophila* female meiosis. Science 273: 118–122. 8658180 10.1126/science.273.5271.118

[B65] Katis VL, Matos J, Mori S, Shirahige K, Zachariae W, Nasmyth K 2004 Spo13 facilitates monopolin recruitment to kinetochores and regulates maintenance of centromeric cohesion during yeast meiosis. Curr Biol 14: 2183–2196. 15620645 10.1016/j.cub.2004.12.020

[B66] Katis VL, Lipp JJ, Imre R, Bogdanova A, Okaz E, Habermann B, Mechtler K, Nasmyth K, Zachariae W 2010 Rec8 phosphorylation by casein kinase 1 and Cdc7–Dbf4 kinase regulates cohesin cleavage by separase during meiosis. Dev Cell 18: 397–409. 20230747 10.1016/j.devcel.2010.01.014 PMC2994640

[B67] Keeney S, Giroux CN, Kleckner N 1997 Meiosis-specific DNA double-strand breaks are catalyzed by Spo11, a member of a widely conserved protein family. Cell 88: 375–384. 9039264 10.1016/s0092-8674(00)81876-0

[B68] Keeney S, Baudat F, Angeles M, Zhou Z-H, Copeland NG, Jenkins NA, Manova K, Jasin M 1999 A mouse homolog of the *Saccharomyces cerevisiae* meiotic recombination DNA transesterase Spo11p. Genomics 61: 170–182. 10534402 10.1006/geno.1999.5956

[B69] Kerrebrock AW, Miyazaki WY, Birnby D, Orr-Weaver TL 1992 The *Drosophila* mei-S332 gene promotes sister-chromatid cohesion in meiosis following kinetochore differentiation. Genetics 130: 827–841. 1582560 10.1093/genetics/130.4.827 PMC1204932

[B70] Kiburz BM, Reynolds DB, Megee PC, Marston AL, Lee BH, Lee TI, Levine SS, Young RA, Amon A 2005 The core centromere and Sgo1 establish a 50-kb cohesin-protected domain around centromeres during meiosis I. Genes Dev 19: 3017–3030. 16357219 10.1101/gad.1373005 PMC1315405

[B71] Kim S, Meyer R, Chuong H, Dawson DS 2013 Dual mechanisms prevent premature chromosome segregation during meiosis. Genes Dev 27: 2139–2146. 24115770 10.1101/gad.227454.113 PMC3850097

[B72] King JM, Nicklas RB 2000 Tension on chromosomes increases the number of kinetochore microtubules but only within limits. J Cell Sci 113: 3815–3823. 11034909 10.1242/jcs.113.21.3815

[B73] Kitajima T, Kawashima S, Watanabe Y 2004 The conserved kinetochore protein shugoshin protects centromeric cohesion during meiosis. Nature 427: 510–517. 14730319 10.1038/nature02312

[B74] Kitajima TS, Sakuno T, Ishiguro K-i, Iemura S-i, Natsume T, Kawashima SA, Watanabe Y 2006 Shugoshin collaborates with protein phosphatase 2A to protect cohesin. Nature 441: 46–52. 16541025 10.1038/nature04663

[B75] Kitamura E, Tanaka K, Komoto S, Kitamura Y, Antony C, Tanaka TU 2009 Kinetochores generate microtubules with distal plus ends: their roles and limited lifetime in mitosis. Dev Cell 18: 248–259. 20159595 10.1016/j.devcel.2009.12.018 PMC2828607

[B76] Koshland DE, Mitchison TJ, Kirschner MW 1988 Polewards chromosome movement driven by microtubule depolymerization in vitro. Nature 331: 499–504. 3340202 10.1038/331499a0

[B77] Kudo NR, Wassmann K, Anger M, Schuh M, Wirth KG, Xu H, Helmhart W, Kudo H, McKay M, Maro B, 2006 Resolution of chiasmata in oocytes requires separase-mediated proteolysis. Cell 126: 135–146. 16839882 10.1016/j.cell.2006.05.033

[B78] LaFountain JR, Cohan CS, Siegel AJ, LaFountain DJ 2004 Direct visualization of microtubule flux during metaphase and anaphase in crane-fly spermatocytes. Mol Biol Cell 15: 5724–5732. 15469981 10.1091/mbc.E04-08-0750 PMC532050

[B79] Lampson MA, Renduchitala K, Khodjakov A, Kapoor TM 2004 Correcting improper chromosome-spindle attachments during cell division. Nat Cell Biol 6: 232–237. 14767480 10.1038/ncb1102

[B80] Lee BH, Amon A 2003 Role of Polo-like kinase CDC5 in programming meiosis I chromosome segregation. Science 300: 482–486. 12663816 10.1126/science.1081846

[B81] Lee BH, Kiburz BM, Amon A 2004 Spo13 maintains centromeric cohesion and kinetochore coorientation during meiosis I. Curr Biol 14: 2168–2182. 15620644 10.1016/j.cub.2004.12.033

[B82] Li X, Dawe RK 2009 Fused sister kinetochores initiate the reductional division in meiosis I. Nat Cell Biol 11: 1103–1108. 19684578 10.1038/ncb1923

[B83] Li X, Nicklas RB 1995 Mitotic forces control a cell-cycle checkpoint. Nature 373: 630–632. 7854422 10.1038/373630a0

[B84] Liu D, Vader G, Vromans MJM, Lampson MA, Lens SMA 2009 Sensing chromosome bi-orientation by spatial separation of Aurora B kinase from kinetochore substrates. Science 323: 1350–1353. 19150808 10.1126/science.1167000 PMC2713345

[B85] Lombillo VA, Stewart RJ, Richard McIntosh J 1995 Minus-end-directed motion of kinesin-coated microspheres driven by microtubule depolymerization. Nature 373: 161–164. 7816099 10.1038/373161a0

[B86] Magidson V, O’Connell Christopher B, Lončarek J, Paul R, Mogilner A, Khodjakov A 2011 The spatial arrangement of chromosomes during prometaphase facilitates spindle assembly. Cell 146: 555–567. 21854981 10.1016/j.cell.2011.07.012 PMC3291198

[B87] Mandelkow E, Mandelkow E 1985 Unstained microtubules studied by cryo-electron microscopy: substructure, supertwist and disassembly. J Mol Biol 181: 123. 3981631 10.1016/0022-2836(85)90330-4

[B88] Mann C, Davis RW 1986 Meiotic disjunction of circular minichromosomes in yeast does not require DNA homology. Proc Natl Acad Sci 83: 6017–6019. 3526347 10.1073/pnas.83.16.6017 PMC386428

[B89] Marston AL 2014 Chromosome segregation in budding yeast: sister chromatid cohesion and related mechanisms. Genetics 196: 31–63. 24395824 10.1534/genetics.112.145144 PMC3872193

[B90] Marston AL, Amon A 2004 Meiosis: cell-cycle controls shuffle and deal. Nat Rev Mol Cell Biol 5: 983–997. 15573136 10.1038/nrm1526

[B91] Marston A, Tham W, Shah H, Amon A 2004 A genome-wide screen identifies genes required for centromeric cohesion. Science 303: 1367–1370. 14752166 10.1126/science.1094220

[B92] Matos J, Lipp JJ, Bogdanova A, Guillot S, Okaz E, Junqueira M, Shevchenko A, Zachariae W 2008 Dbf4-dependent Cdc7 kinase links DNA replication to the segregation of homologous chromosomes in meiosis I. Cell 135: 662–678. 19013276 10.1016/j.cell.2008.10.026

[B93] Megee PC, Mistrot C, Guacci V, Koshland D 1999 The centromeric sister chromatid cohesion site directs Mcd1p binding to adjacent sequences. Mol Cell 4: 445–450. 10518226 10.1016/s1097-2765(00)80347-0

[B94] Meyer RE, Kim S, Obeso D, Straight PD, Winey M, Dawson DS 2013 Mps1 and Ipl1/Aurora B act sequentially to correctly orient chromosomes on the meiotic spindle of budding yeast. Science 339: 1071–1074. 23371552 10.1126/science.1232518 PMC3604795

[B95] Miller MP, Ünal E, Brar GA, Amon A 2012 Meiosis I chromosome segregation is established through regulation of microtubule–kinetochore interactions. eLife 1: e00117. 23275833 10.7554/eLife.00117 PMC3525924

[B96] Miranda JL, Wulf PD, Sorger PK, Harrison SC 2005 The yeast DASH complex forms closed rings on microtubules. Nat Struct Mol Biol 12: 138–143. 15640796 10.1038/nsmb896

[B97] Monje-Casas F, Prabhu VR, Lee BH, Boselli M, Amon A 2007 Kinetochore orientation during meiosis is controlled by Aurora B and the monopolin complex. Cell 128: 477–490. 17289568 10.1016/j.cell.2006.12.040 PMC1808280

[B98] Newnham L, Jordan P, Rockmill B, Roeder GS, Hoffmann E 2010 The synaptonemal complex protein, Zip1, promotes the segregation of nonexchange chromosomes at meiosis I. Proc Natl Acad Sci 107: 781–785. 20080752 10.1073/pnas.0913435107 PMC2818913

[B99] Ng TM, Waples WG, Lavoie BD, Biggins S 2009 Pericentromeric sister chromatid cohesion promotes kinetochore biorientation. Mol Biol Cell 20: 3818–3827. 19605555 10.1091/mbc.E09-04-0330 PMC2735481

[B100] Nicklas RB 1965 Chromosome velocity during mitosis as a function of chromosome size and position. J Cell Biol 25: 119–135. 14342826 10.1083/jcb.25.1.119 PMC2106602

[B101] Nicklas RB 1983 Measurements of the force produced by the mitotic spindle in anaphase. J Cell Biol 97: 542–548. 6885908 10.1083/jcb.97.2.542 PMC2112533

[B102] Nicklas R 1989 The motor for poleward chromosome movement in anaphase is in or near the kinetochore. J Cell Biol 109: 2245–2255. 2808528 10.1083/jcb.109.5.2245 PMC2115846

[B103] Nicklas RB 1997 How cells get the right chromosomes. Science 275: 632–637. 9005842 10.1126/science.275.5300.632

[B104] Nicklas RB, Ward SC, Gorbsky GJ 1995 Kinetochore chemistry is sensitive to tension and may link mitotic forces to a cell cycle checkpoint. J Cell Biol 130: 929–939. 7642708 10.1083/jcb.130.4.929 PMC2199958

[B105] Noda Y, Sato-Yoshitake R, Kondo S, Nangaku M, Hirokawa N 1995 KIF2 is a new microtubule-based anterograde motor that transports membranous organelles distinct from those carried by kinesin heavy chain or KIF3A/B. J Cell Biol 129: 157. 7535303 10.1083/jcb.129.1.157 PMC2120367

[B106] Page J, Viera A, Parra MT, de la Fuente R, Suja JÁ, Prieto I, Barbero JL, Rufas JS, Berríos S, Fernández-Donoso R 2006 Involvement of synaptonemal complex proteins in sex chromosome segregation during marsupial male meiosis. PLoS Genet 2: e136. 16934004 10.1371/journal.pgen.0020136 PMC1557784

[B107] Paliulis LV, Nicklas RB 2000 The reduction of chromosome number in meiosis is determined by properties built into the chromosomes. J Cell Biol 150: 1223–1232. 10995430 10.1083/jcb.150.6.1223 PMC2150703

[B108] Parra MT, Viera A, Gómez R, Page J, Benavente R, Santos JL, Rufas JS, Suja JA 2004 Involvement of the cohesin Rad21 and SCP3 in monopolar attachment of sister kinetochores during mouse meiosis I. J Cell Sci 117: 1221–1234. 14970259 10.1242/jcs.00947

[B109] Peplowska K, Wallek AU, Storchova Z 2014 Sgo1 regulates both condensin and Ipl1/Aurora B to promote chromosome biorientation. PLoS Genet 10: e1004411. 24945276 10.1371/journal.pgen.1004411 PMC4063673

[B110] Petronczki M, Matos J, Mori S, Gregan J, Bogdanova A, Schwickart M, Mechtler K, Shirahige K, Zachariae W, Nasmyth K 2006 Monopolar attachment of sister kinetochores at meiosis I requires casein kinase 1. Cell 126: 1049–1064. 16990132 10.1016/j.cell.2006.07.029

[B111] Powers AF, Franck AD, Gestaut DR, Cooper J, Gracyzk B, Wei RR, Wordeman L, Davis TN, Asbury CL 2009 The Ndc80 kinetochore complex forms load-bearing attachments to dynamic microtubule tips via biased diffusion. Cell 136: 865–875. 19269365 10.1016/j.cell.2008.12.045 PMC2749323

[B112] Rabitsch KP, Petronczki M, Javerzat J-P, Genier S, Chwalla B, Schleiffer A, Tanaka TU, Nasmyth K 2003 Kinetochore recruitment of two nucleolar proteins is required for homolog segregation in meiosis I. Dev Cell 4: 535–548. 12689592 10.1016/s1534-5807(03)00086-8

[B113] Rabitsch KP, Gregan J, Schleiffer A, Javerzat J-P, Eisenhaber F, Nasmyth K 2004 Two fission yeast homologs of *Drosophila* Mei-S332 are required for chromosome segregation during meiosis I and II. Curr Biol 14: 287–301. 14972679 10.1016/j.cub.2004.01.051

[B114] Rasmussen S 1977 The transformation of the synaptonemal complex into the ‘elimination chromatin’ in *Bombyx mori* oocytes. Chromosoma 60: 205–221. 870294 10.1007/BF00329771

[B115] Ribeiro SA, Gatlin JC, Dong Y, Joglekar A, Cameron L, Hudson DF, Farr CJ, McEwen BF, Salmon ED, Earnshaw WC, 2009 Condensin regulates the stiffness of vertebrate centromeres. Mol Biol Cell 20: 2371–2380. 19261808 10.1091/mbc.E08-11-1127 PMC2675617

[B116] Riedel CG, Katis VL, Katou Y, Mori S, Itoh T, Helmhart W, Gálová M, Petronczki M, Gregan J, Cetin B, 2006 Protein phosphatase 2A protects centromeric sister chromatid cohesion during meiosis I. Nature 441: 53–61. 16541024 10.1038/nature04664

[B117] Rieder CL, Alexander SP 1990 Kinetochores are transported poleward along a single astral microtubule during chromosome attachment to the spindle in newt lung cells. J Cell Biol 110: 81–95. 2295685 10.1083/jcb.110.1.81 PMC2115982

[B118] Romanienko PJ, Camerini-Otero RD 1999 Cloning, characterization, and localization of mouse and human SPO11. Genomics 61: 156–169. 10534401 10.1006/geno.1999.5955

[B119] Sakuno T, Tada K, Watanabe Y 2009 Kinetochore geometry defined by cohesion within the centromere. Nature 458: 852–858. 19370027 10.1038/nature07876

[B120] Sakuno T, Tanaka K, Hauf S, Watanabe Y 2011 Repositioning of Aurora B promoted by chiasmata ensures sister chromatid mono-orientation in meiosis I. Dev Cell 21: 534–545. 21920317 10.1016/j.devcel.2011.08.012

[B121] Sarangapani KK, Akiyoshi B, Duggan NM, Biggins S, Asbury CL 2013 Phosphoregulation promotes release of kinetochores from dynamic microtubules via multiple mechanisms. Proc Natl Acad Sci 110: 7282–7287. 23589891 10.1073/pnas.1220700110 PMC3645574

[B122] Sarangapani KK, Duro E, Deng Y, Alves Fd L, Ye Q, Opoku KN, Ceto S, Rappsilber J, Corbett KD, Biggins S, 2014 Sister kinetochores are mechanically fused during meiosis I in yeast. Science 346: 248–251. 25213378 10.1126/science.1256729 PMC4226495

[B123] Sarkar S, Shenoy RT, Dalgaard JZ, Newnham L, Hoffmann E, Millar JBA, Arumugam P 2013 Monopolin subunit Csm1 associates with MIND complex to establish monopolar attachment of sister kinetochores at meiosis I. PLoS Genet 9: e1003610. 23861669 10.1371/journal.pgen.1003610 PMC3701701

[B124] Schuh M, Ellenberg J 2007 Self-organization of MTOCs replaces centrosome function during acentrosomal spindle assembly in live mouse oocytes. Cell 130: 484–498. 17693257 10.1016/j.cell.2007.06.025

[B125] Schvarzstein M, Wignall SM, Villeneuve AM 2010 Coordinating cohesion, co-orientation, and congression during meiosis: lessons from holocentric chromosomes. Genes Dev 24: 219–228. 20123904 10.1101/gad.1863610 PMC2811823

[B126] Severson AF, Ling L, van Zuylen V, Meyer BJ 2009 The axial element protein HTP-3 promotes cohesin loading and meiotic axis assembly in *C. elegans* to implement the meiotic program of chromosome segregation. Genes Dev 23: 1763–1778. 19574299 10.1101/gad.1808809 PMC2720254

[B127] Shonn MA, McCarroll R, Murray AW 2002 Spo13 protects meiotic cohesin at centromeres in meiosis I. Genes Dev 16: 1659–1671. 12101124 10.1101/gad.975802 PMC186364

[B128] Song Y, Mandelkow E 1993 Recombinant kinesin motor domain binds to β-tubulin and decorates microtubules with a B surface lattice. Proc Natl Acad Sci 90: 1671. 8446580 10.1073/pnas.90.5.1671 PMC45941

[B129] Stephens AD, Haase J, Vicci L, Taylor RM, Bloom K 2011 Cohesin, condensin, and the intramolecular centromere loop together generate the mitotic chromatin spring. J Cell Biol 193: 1167–1180. 21708976 10.1083/jcb.201103138 PMC3216333

[B130] Stephens AD, Haggerty RA, Vasquez PA, Vicci L, Snider CE, Shi F, Quammen C, Mullins C, Haase J, Taylor RM, 2013 Pericentric chromatin loops function as a nonlinear spring in mitotic force balance. J Cell Biol 200: 757–772. 23509068 10.1083/jcb.201208163 PMC3601350

[B131] Tada K, Susumu H, Sakuno T, Watanabe Y 2011 Condensin association with histone H2A shapes mitotic chromosomes. Nature 474: 477–483. 21633354 10.1038/nature10179

[B132] Tanaka T, Cosma MP, Wirth K, Nasmyth K 1999 Identification of cohesin association sites at centromeres and along chromosome arms. Cell 98: 847–858. 10499801 10.1016/s0092-8674(00)81518-4

[B133] Tanaka T, Fuchs J, Loidl J, Nasmyth K 2000 Cohesin ensures bipolar attachment of microtubules to sister centromeres and resists their precocious separation. Nat Cell Biol 2: 492–499. 10934469 10.1038/35019529

[B134] Tanaka TU, Rachidi N, Janke C, Pereira G, Galova M, Schiebel E, Stark MJR, Nasmyth K 2002 Evidence that the Ipl1–Sli15 (Aurora kinase–INCENP) complex promotes chromosome bi-orientation by altering kinetochore–spindle pole connections. Cell 108: 317–329. 11853667 10.1016/s0092-8674(02)00633-5

[B135] Tanaka K, Mukae N, Dewar H, van Breugel M, James EK, Prescott AR, Antony C, Tanaka TU 2005 Molecular mechanisms of kinetochore capture by spindle microtubules. Nature 434: 987–994. 15846338 10.1038/nature03483

[B136] Tanaka K, Kitamura E, Kitamura Y, Tanaka TU 2007 Molecular mechanisms of microtubule-dependent kinetochore transport toward spindle poles. J Cell Biol 178: 269–281. 17620411 10.1083/jcb.200702141 PMC2064446

[B137] Tanaka K, Li Chang H, Kagami A, Watanabe Y 2009 CENP-C functions as a scaffold for effectors with essential kinetochore functions in mitosis and meiosis. Dev Cell 17: 334–343. 19758558 10.1016/j.devcel.2009.08.004

[B138] Thomas SE, Soltani-Bejnood M, Roth P, Dorn R, Logsdon JM Jr, McKee BD 2005 Identification of two proteins required for conjunction and regular segregation of achiasmate homologs in *Drosophila* male meiosis. Cell 123: 555–568. 16286005 10.1016/j.cell.2005.08.043

[B139] Tóth A, Rabitsch KP, Gálová M, Schleiffer A, Buonomo SBC, Nasmyth K 2000 Functional genomics identifies monopolin: a kinetochore protein required for segregation of homologs during meiosis I. Cell 103: 1155–1168. 11163190 10.1016/s0092-8674(00)00217-8

[B140] Tsai J-H, Yan R, McKee B 2011 Homolog pairing and sister chromatid cohesion in heterochromatin in *Drosophila* male meiosis I. Chromosoma 120: 335–351. 21384262 10.1007/s00412-011-0314-0

[B141] Tsubouchi T, Roeder GS 2005 A synaptonemal complex protein promotes homology-independent centromere coupling. Science 308: 870–873. 15879219 10.1126/science.1108283

[B142] Tytell JD, Sorger PK 2006 Analysis of kinesin motor function at budding yeast kinetochores. J Cell Biol 172: 861–874. 16533946 10.1083/jcb.200509101 PMC2063730

[B143] Umbreit NT, Gestaut DR, Tien JF, Vollmar BS, Gonen T, Asbury CL, Davis TN 2012 The Ndc80 kinetochore complex directly modulates microtubule dynamics. Proc Natl Acad Sci 109: 16113–16118. 22908300 10.1073/pnas.1209615109 PMC3479545

[B144] Verzijlbergen KF, Nerusheva OO, Kelly D, Kerr A, Clift D, de Lima Alves F, Rappsilber J, Marston AL 2014 Shugoshin biases chromosomes for biorientation through condensin recruitment to the pericentromere. eLife 3: e01374. 24497542 10.7554/eLife.01374 PMC3910079

[B145] Watanabe Y, Nurse P 1999 Cohesin Rec8 is required for reductional chromosome segregation at meiosis. Nature 400: 461–464. 10440376 10.1038/22774

[B146] Welburn JPI, Vleugel M, Liu D, Yates Iii JR, Lampson MA, Fukagawa T, Cheeseman IM 2010 Aurora B phosphorylates spatially distinct targets to differentially regulate the kinetochore–microtubule interface. Mol Cell 38: 383–392. 20471944 10.1016/j.molcel.2010.02.034 PMC2873218

[B147] Westermann S, Schleiffer A 2013 Family matters: structural and functional conservation of centromere-associated proteins from yeast to humans. Trends Cell Biol 23: 260–269. 23481674 10.1016/j.tcb.2013.01.010

[B148] Westermann S, Avila-Sakar A, Wang H-W, Niederstrasser H, Wong J, Drubin DG, Nogales E, Barnes G 2005 Formation of a dynamic kinetochore–microtubule interface through assembly of the Dam1 ring complex. Mol Cell 17: 277–290. 15664196 10.1016/j.molcel.2004.12.019

[B149] Westermann S, Wang H-W, Avila-Sakar A, Drubin DG, Nogales E, Barnes G 2006 The Dam1 kinetochore ring complex moves processively on depolymerizing microtubule ends. Nature 440: 565–569. 16415853 10.1038/nature04409

[B150] Winey M, Morgan GP, Straight PD, Giddings TH, Mastronarde DN 2005 Three-dimensional ultrastructure of *Saccharomyces cerevisiae* meiotic spindles. Mol Biol Cell 16: 1178–1188. 15635095 10.1091/mbc.E04-09-0765 PMC551483

[B151] Wolf KW 1994 How meiotic cells deal with non-exchange chromosomes. BioEssays 16: 107–114. 8147841 10.1002/bies.950160207

[B152] Yang Z, Tulu US, Wadsworth P, Rieder CL 2007 Kinetochore dynein is required for chromosome motion and congression independent of the spindle checkpoint. Curr Biol 17: 973–980. 17509882 10.1016/j.cub.2007.04.056 PMC2570756

[B153] Yi K, Rubinstein B, Li R 2013 Symmetry breaking and polarity establishment during mouse oocyte maturation. Philos Trans R Soc Lond B Biol Sci 368: 20130002. 24062576 10.1098/rstb.2013.0002 PMC3785956

[B154] Yokobayashi S, Watanabe Y 2005 The kinetochore protein Moa1 enables cohesion-mediated monopolar attachment at meiosis I. Cell 123: 803–817. 16325576 10.1016/j.cell.2005.09.013

[B155] Yong-Gonzalez V, Wang B-D, Butylin P, Ouspenski I, Strunnikov A 2007 Condensin function at centromere chromatin facilitates proper kinetochore tension and ensures correct mitotic segregation of sister chromatids. Genes Cells 12: 1075–1090. 17825050 10.1111/j.1365-2443.2007.01109.x PMC2674963

[B156] Zickler D, Kleckner N 1999 Meiotic chromosomes: integrating structure and function. Annu Rev Genet 33: 603–754. 10690419 10.1146/annurev.genet.33.1.603

